# Genetic diversity and fingerprinting of 33 standard flue-cured tobacco varieties for use in distinctness, uniformity, and stability testing

**DOI:** 10.1186/s12870-020-02596-w

**Published:** 2020-08-17

**Authors:** Binbin He, Ruimei Geng, Lirui Cheng, Xianbin Yang, Hongmei Ge, Min Ren

**Affiliations:** 1 Key Laboratory of Tobacco Improvement and Biotechnology, Tobacco Research Institute of Chinese Academy of Agricultural Sciences, No.11 Keyuanjingsi Road, Qingdao, 266100 Shandong China; 2 Technical Center of Zunyi Branch Company of Guizhou Tobacco Company, Zunyi, 563000 Guizhou China; 3 Qingdao Academy of Agricultural Sciences, Qingdao, 266100 Shandong China

**Keywords:** Tobacco, DUS testing, Genetic fingerprinting, Genetic diversity

## Abstract

**Background:**

At present, the distinctness, uniformity, and stability (DUS) testing of flue-cured tobacco (*Nicotiana tabacum* L.) depends on field morphological identification, which is problematic in that it is labor intensive, time-consuming, and susceptible to environmental impacts. In order to improve the efficiency and accuracy of tobacco DUS testing, the development of a molecular marker-based method for genetic diversity identification is urgently needed.

**Results:**

In total, 91 simple sequence repeats (SSR) markers with clear and polymorphic amplification bands were obtained with polymorphism information content, Nei index, and Shannon information index values of 0.3603, 0.4040, and 0.7228, respectively. Clustering analysis showed that the 33 study varieties, which are standard varieties for flue-cured tobacco DUS testing, could all be distinguished from one another. Further analysis showed that a minimum of 25 markers were required to identify the genetic diversity of these varieties. Following the principle of two markers per linkage group, 48 pairs of SSR markers were selected. Correlation analysis showed that the genetic relationships revealed by the 48 SSR markers were consistent with those found using the 91 SSR markers.

**Conclusions:**

The genetic fingerprints of the 33 standard varieties of flue-cured tobacco were constructed using 48 SSR markers, and an SSR marker-based identification technique for new tobacco varieties was developed. This study provides a reliable technological approach for determining the novelty of new tobacco varieties and offers a solid technical basis for the accreditation and protection of new tobacco varieties.

## Background

New plant varieties are needed to increase agricultural production and efficiency. The protection of intellectual property rights for new plant varieties is a well-established practice and is also a symbol of progress in human civilizations [1]. The protection of new plant varieties cannot be realized without the support of a series of technical conditions. Distinctness, uniformity, and stability (DUS) are three technical and scientific criteria for the protection of new plant varieties [2, 3]. In 1999, China officially joined the Convention on the Protection of New Plant Varieties and became a member of the International Union for the Protection of New Varieties of Plants (UPOV). Using the UPOV DUS testing guidelines as an example, China developed a series of crop DUS testing guidelines and promoted the use of these guidelines in the protection of new plant varieties [3]. In 2002, the UPOV released the first DUS testing guidelines for tobacco (*Nicotiana tabacum* L.) [4]. Thereafter, China developed and released the first domestic tobacco DUS testing standard, the Guidelines for the Conduct of Tests for Distinctness, Uniformity, Stability – Flue-Cured Tobacco (*Nicotiana tabacum* L.; YC/T 369–2010) [5], which was based on the General Directives for the Conduct of Tests of Distinctness, Uniformity, Stability for New Varieties of Plants (GB/T 19557.1–2004) [6] and the tobacco testing guidelines of the UPOV [4].

DUS testing is a complex technical process [2, 7]. Currently, the domestic and foreign DUS testing standards for new plant varieties are mainly based on field measurements of biological, agronomic, quality, and resistance traits. For example, the Chinese DUS testing guidelines for flue-cured tobacco include 35 basic measurement traits, of which 16 traits must be mandatorily measured [5]. Of all the measured characteristics, differences with regard to either one quality character or two quantity characters among the candidate and approximate varieties are used to judge the distinctness. To assess the uniformity of a population, a standard of 1% with an acceptance probability of at least 95% should be applied. To assess the stability of a candidate variety, at least two planting seasons should be evaluated [4, 5].

Given that DUS testing is based on the apparent morphological characteristics of the study plants, the results and comparative analysis of candidate, standard, and approximate varieties will be influenced by environmental factors [8]. In addition, different testers may subjectively perceive traits differently, leading to inconsistencies in the evaluation of certain traits [9]. Moreover, the substantial workload involved further increases the likelihood of human error in DUS testing. The application of molecular marker-based technologies for the identification of plant varieties has several advantages over traditional DUS testing methods, including rapid processing times, an immunity to the influence of environmental factors, and easy automation [10]. Therefore, molecular marker-based methods represent an emerging trend in rapid DUS testing [2, 7, 11–13]. Of the numerous molecular marker technologies available, simple sequence repeats (SSR) analysis is considered ideal for the DUS testing of new varieties [8, 14–18] and the fingerprinting of standard crop varieties [10–12] due to multiple associated advantages, such as the abundance, high polymorphism, and co-dominance of SSR markers [19, 20] and the stability, repeatability, and simple operational procedures involved in SSR analysis [10, 21, 22].

In the present study, we addressed the lack of molecular marker-based technologies for estimating the distinctness, uniformity, and stability of flue-cured tobacco varieties by carrying out a population genetics study and constructing SSR fingerprints of 33 standard flue-cured tobacco varieties that are commonly used in DUS testing [5]. Thus, we developed an identification method to distinguish tobacco varieties that provides a technological basis for the identification and protection of new flue-cured tobacco varieties.

## Results

### Genetic diversity analysis

**Fig. 1:**
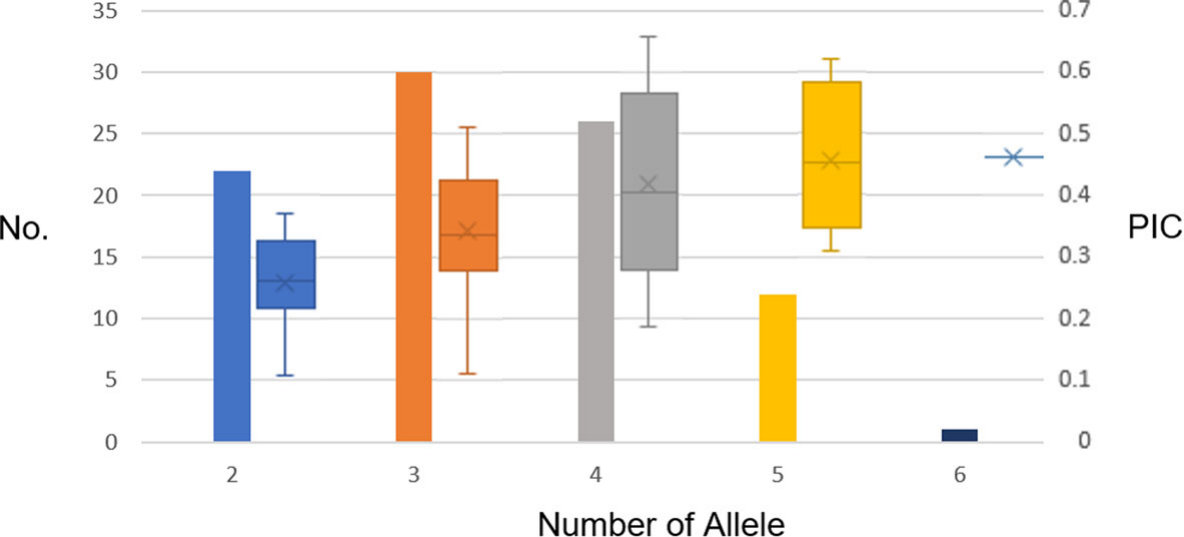
Number and PIC of SSRs with different allele numbers. The primary axis is the number of SSRs, represented by the histogram in the diagram, and the secondary axis shows the PIC values, represented by the boxes

**Fig. 2:**
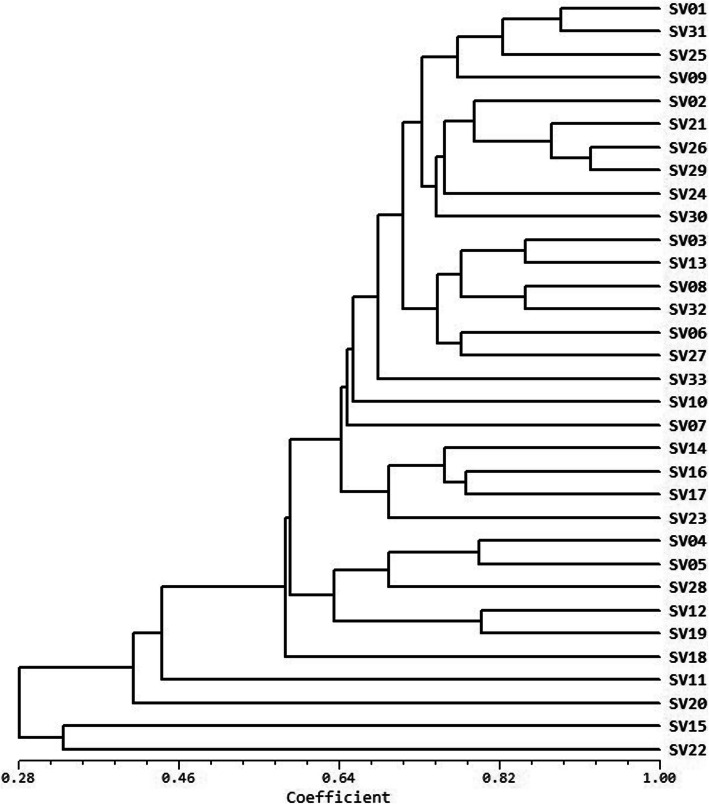
UPGMA clustering tree of the 33 flue-cured tobacco varieties, all of which could be fully distinguished from one another

The amplification of 270 SSR marker candidates led to the selection of 91 pairs of polymorphic SSR loci with clear amplified bands (Additional file 1: Table S1). The examination of these 91 SSR loci in the 33 standard varieties revealed 304 alleles (2–6 alleles per locus) and an average of 3.34 alleles per locus. These alleles included 67 rare alleles with allele frequencies ≤0.05. The SSR loci with 4 or 5 alleles also had the highest number of rare alleles, 28 and 22 rare alleles, respectively. These rare alleles accounted for 75% of the total number of rare alleles. No rare alleles were detected in loci with 2 alleles. The polymorphic information content (PIC), Nei index (H), and Shannon information index (I) values of the 91 SSR pairs were 0.3603, 0.4040, and 0.7228, respectively. A boxplot of the PIC values by allele number revealed that the polymorphism of a given locus increased with the number of alleles (Fig. 1). Cluster analysis showed that the average genetic similarity between varieties was 0.5640 ± 0.1744. According to the unweighted pair group method with arithmetic mean (UPGMA) clustering tree, the 33 standard varieties can be fully distinguished from one another using 91 pairs of SSR markers (Fig. 2).

### Evaluation of the minimum number of primers required for genetic diversity analysis

**Fig. 3:**
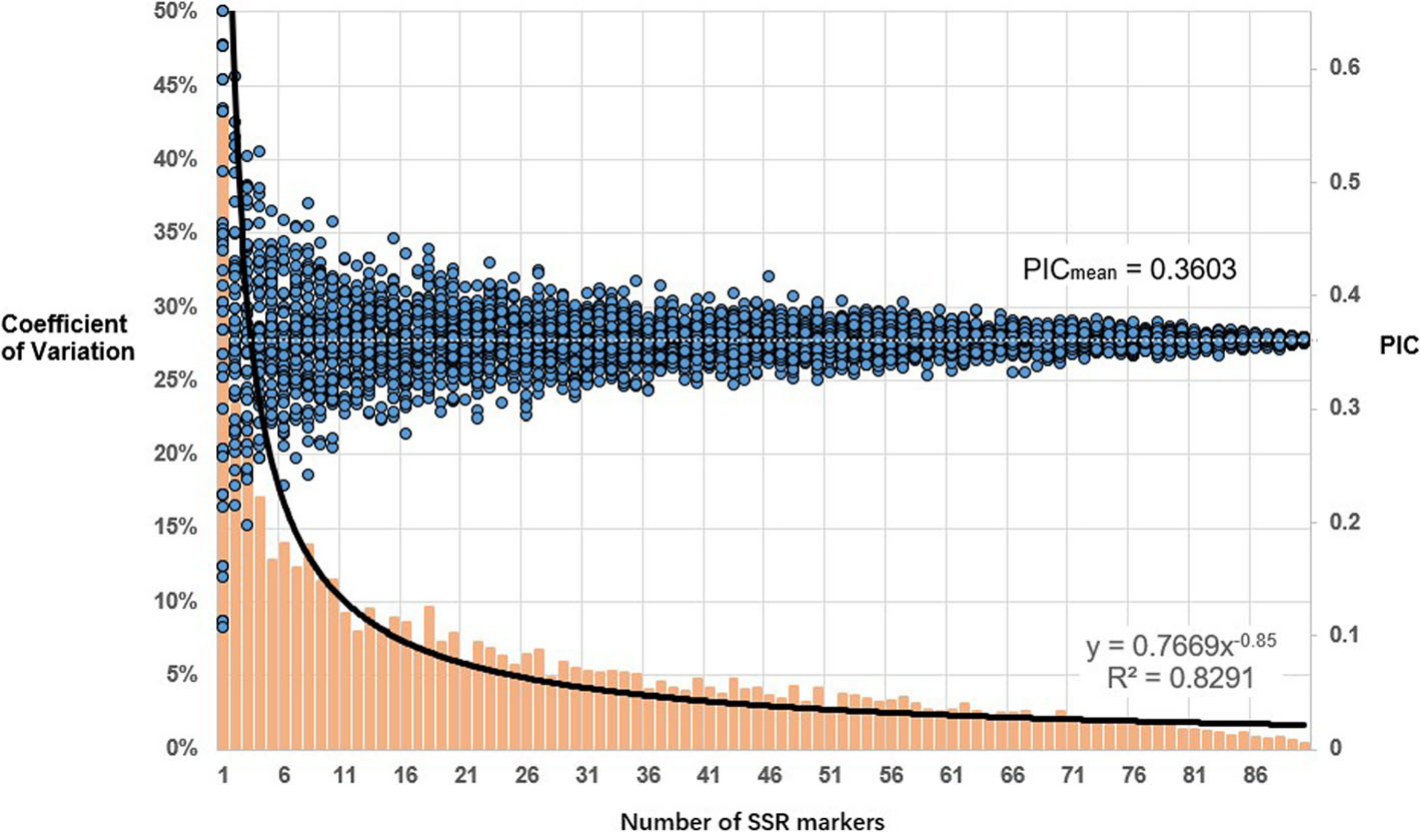
The PIC value and the CV of the sampling experiments for different SSR marker numbers. The *x*-axis shows the number of SSR markers. The primary *y*-axis (left) shows the CV of the PIC value, which is plotted as a histogram. The secondary *y*-axis (right) shows the PIC value of each sample, which is plotted as a scatter plot. As the number of markers increases, the points in the scatter plot (representing the PIC values) tended towards the mean PIC value, and the CV between samples became smaller

To evaluate the minimum number of primers required for genetic diversity analysis, we analyzed how the measured genetic diversity varied with the number of primers. From 1 marker to 90 markers, the random sampling test of each marker number was repeated 50 times, and the average PIC values of each marker number were calculated. A scatter plot of the results revealed that PIC values gradually tend towards the average PIC value as the number of markers increases (Fig. 3). Thus, using more markers decreases the coefficient of variation (CV) between repeats, as the histogram at the bottom of Fig. 3 shows. By calculating the CV trend line, we found that using more than 25 markers resulted in a CV < 5.0%, indicating that the PIC values were stable. Therefore, a subset of 25 markers (out of the 91 markers tested in this study) is sufficient to reveal the genetic diversity of a population.

### The use of SSR marker genotyping to construct the genetic fingerprints of the studied varieties

**Fig. 4:**
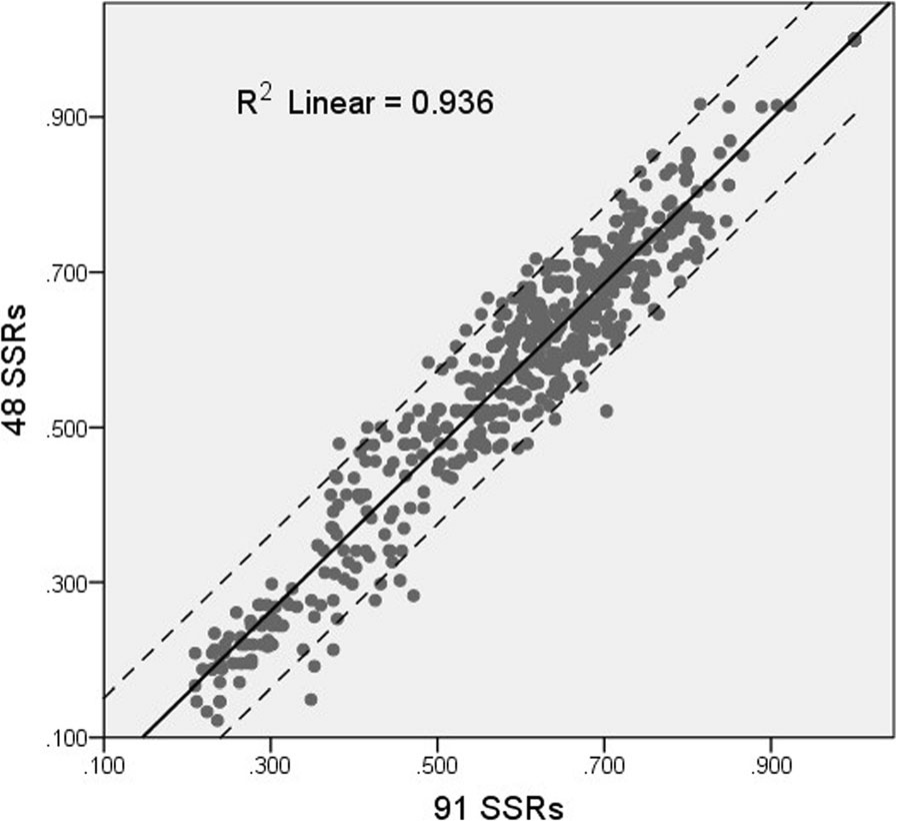
The genetic similarity matrices of the 48 and 91 SSR markers were calculated and their correlation is displayed as a scatter plot. The dotted range shows the 95% confidence intervals. The genetic relationships revealed by the two sets of markers were significantly correlated

Following the principle of using two markers for each linkage group, we selected 48 pairs of SSR markers from the 91 markers tested to be used for the construction of the genetic fingerprints of the standard flue-cured tobacco varieties commonly used in DUS testing. The PIC, H, and I values of the 48 markers were 0.3736, 0.4223, and 0.7534, respectively. Using the 48 pairs not only met the requirements for the minimum number of primers but were also sufficient to fully distinguish the 33 varieties from one another. Furthermore, we calculated and plotted genetic similarity matrix to compare the differences in the genetic relationships revealed by the 48 and 91 markers selected. The points in the scatter plot are arranged along a diagonal line with significant linearity, all within the 95% confidence interval of the linear fit. Subsequent correlation analysis revealed a significant correlation between the genetic relationships determined by the two sets of markers, with a Pearson correlation coefficient of 0.967 (Fig. 4).

### Construction of SSR genetic fingerprints of the 33 standard varieties

**Fig. 5:**
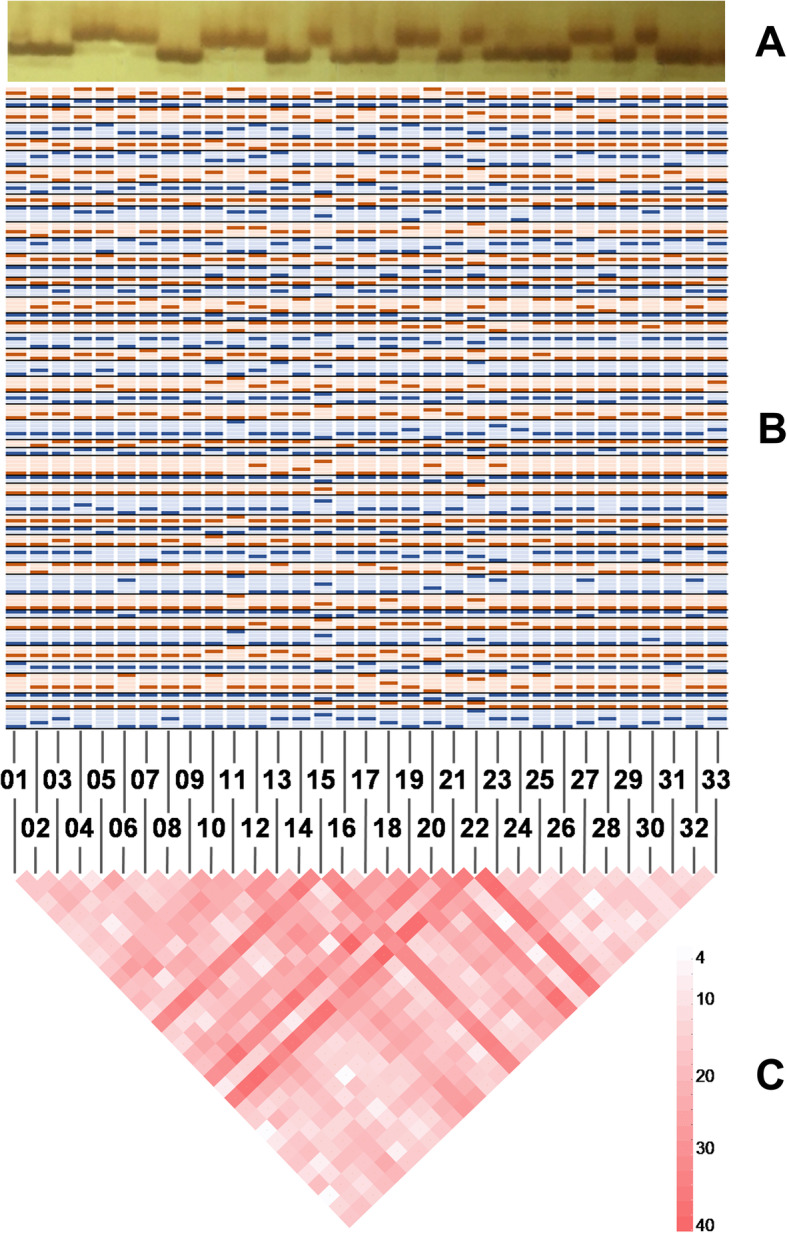
**a** The electrophoretic photo of SSR marker PT50136 (The original electrophoretic image is shown on the right side of Additional file 2: Figure S1). **b** The fingerprint band pattern of the 33 standard varieties constructed using 48 pairs of SSR markers. The band pattern is arranged alternately in blue and orange to distinguish markers, and each column represents a variety. **c** The triangular matrix of differentiated locus number among the studied varieties

The genetic fingerprints of the 33 standard varieties were constructed using 48 pairs of SSR markers and produced the banding patterns shown in Fig. 5a-b. The fingerprints contained 162 alleles with allele frequencies that ranged from 0.0303 to 0.9394 and an average allele frequency of 0.2963 ± 0.2897. There were 39 rare alleles with allele frequencies ≤0.05. Eleven of the varieties carried a rare allele, the varieties SV15, SV22, SV11, and SV20 contained 15, 7, 6, and 4 rare alleles, respectively. The number of differentiated loci among the tested varieties ranged from 4 to 40, with an average of 20.15 ± 7.716. Figure 5c shows that SV22, SV15, and SV20 have more differentiated loci than the other varieties, indicating that they are exceptionally different.

### Core SSR markers for molecular DUS testing of flue-cured tobacco

The 48 SSR pairs revealed that there were at least four differentiated loci among all varieties. Therefore, this set of markers can be used for molecular DUS testing of new varieties of flue-cured tobacco. As such, we screened reference varieties for each allele according to the PCR band pattern. We selected 16 varieties to be used as reference varieties: SV02, SV03, SV04, SV08, SV10, SV11, SV12, SV14, SV15, SV18, SV19, SV20, SV22, SV23, SV30, and SV32. These 16 varieties each had typical and clear amplified bands for a specific allele. In DUS testing that employs the 48 pairs of SSR markers, these varieties can be added as a reference to evaluate the banding patterns of candidate varieties according to the results presented in Table 1.

**Table 1:** Basic information, allele variation, and reference varieties of the 48 selected SSR markers

SSRs	Group	Genetic position (cM)	Allele number	Allele variation and the reference varieties				
				1	2	3	4	5
PT54339	1	6.654	3	SV08	SV19	SV23	–	–
PT50862	1	98.775	3	SV14	SV08	SV15	–	–
PT53216	2	0	3	SV11	SV08	SV30	–	–
PT52432	2	53.864	3	SV08	SV20	SV10	–	–
PT53362	3	45.272	3	SV10	SV08	SV15	–	–
PT60080	3	179.15	4	SV10	SV15	SV12	SV11	–
PT53970	4	54.25	2	SV08	SV15	–	–	–
PT51682	4	76.882	4	SV14	SV10	SV20	SV11	–
PT51072	5	67.324	3	SV15	SV08	SV03	–	–
PT61414	5	79.527	3	SV15	SV10	SV23	–	–
PT60038	6	11.242	3	SV22	SV15	SV08	–	–
PT50434	6	96.873	4	SV12	SV03	SV10	SV08	–
PT50599	7	37.82	5	SV22	SV15	SV12	SV14	SV08
PT60435	7	115.365	4	SV11	SV10	SV12	SV08	–
PT50668	8	1.099	2	SV08	SV15	–	–	–
PT61279	8	120.597	3	SV15	SV12	SV08	–	–
PT50280	9	4.97	4	SV08	SV22	SV10	SV15	–
PT60917	9	38.417	2	SV15	SV08	–	–	–
PT51144	10	24.533	4	SV08	SV12	SV15	SV19	–
PT54061	10	46.603	2	SV08	SV15	–	–	–
PT51398	11	2.208	4	SV22	SV11	SV08	SV02	–
PT54027	11	50.95	2	SV10	SV22	–	–	–
PT51896	12	130.061	4	SV11	SV22	SV08	SV15	–
PT60934	12	55.632	5	SV22	SV15	SV08	SV02	SV10
PT53568	13	38.293	4	SV14	SV03	SV08	SV10	–
PT60844	13	75.285	4	SV11	SV15	SV22	SV08	–
PT61499	14	0	4	SV15	SV20	SV14	SV22	–
PT54448	14	42.076	2	SV08	SV10	–	–	–
PT30201	15	64.96	5	SV11	SV23	SV19	SV08	SV15
PT54772	15	108.802	3	SV10	SV08	SV23	–	–
PT20275	16	23.212	3	SV11	SV15	SV08	–	–
PT55150	16	76.1	4	SV32	SV08	SV22	SV23	–
PT50748	17	12.738	4	SV22	SV10	SV23	SV08	–
PT50693	17	20.14	4	SV08	SV12	SV10	SV15	–
PT51059	18	15.309	3	SV22	SV15	SV08	–	–
PT60742	18	40.185	4	SV11	SV22	SV15	SV08	–
PT50500	19	96.118	3	SV10	SV08	SV12	–	–
PT54889	19	106.147	4	SV15	SV08	SV10	SV03	–
PT50298	20	78.404	3	SV08	SV23	SV10	–	–
PT30421	20	93.545	2	SV08	SV23	–	–	–
PT51951	21	41.966	5	SV23	SV22	SV18	SV08	SV20
PT51289	21	49.7	3	SV20	SV08	SV15	–	–
PT51152	22	96.25	5	SV22	SV15	SV04	SV10	SV08
PT52041	22	142.487	3	SV12	SV08	SV15	–	–
PT50336	23	24.858	2	SV10	SV08	–	–	–
PT50136	23	69.205	2	SV10	SV08	–	–	–
PT50541	24	40.707	5	SV11	SV23	SV15	SV20	SV08
PT52828	24	69.018	2	SV10	SV08	–	–	–

## Discussion

In this study, we used a population of standard flue-cured tobacco varieties that are commonly used in DUS testing and amplified and evaluated marker loci that were selected from a high-density SSR genetic linkage map for tobacco. Analysis of the genetic diversity of these varieties revealed that PIC, H, and I values were 0.3603, 0.4040, and 0.7228, respectively. These values are higher than those presented in studies by Fan et al. (PIC = 0.299) [23], Zheng et al. (I = 0.6567) [24], and Dai et al. (PIC = 0.343) [25], which were based on the same genetic map. However, our results were slightly lower than those of Fricano et al. [26] and Xu et al. [27], which is probably because the populations evaluated by Fricano et al. [26] and Xu et al. [27] not only included flue-cured tobacco but also numerous other varieties. Overall, the DUS testing standard varieties are representative of the phenotypic and genetic variation in flue-cured tobacco. Therefore, these varieties can be used for genetic studies and to construct a technical system for the identification of flue-cured tobacco varieties.

A reasonable evaluation of the genetic diversity of a population requires sufficient genetic markers [28, 29].The studies of minimum number of primers were carried out in different species, such as wheat (*Triticum aestivum* L.) [30], soybean [*Glycine max* (L.) Merr.] [31], wild rice (*Oryza rufipogon* Griff.) [32], and rice (*Oryza sativa* L.) [33, 34]. Although our aim was to reveal the genetic differences among tobacco varieties, we also tried to reduce the number of markers needed in order to keep costs low and improve the detection efficiency. We found that the varieties evaluated in this study can be fully distinguished from one another using 91 pairs of SSR markers, and the genetic diversity of the varieties was similar to or slightly higher than that of other studies. We then tried to reduce the number of primers through repeated random subsampling and a comparison of genetic diversity coefficients. The simulation showed that a subset of only 25 pairs of SSR markers was necessary to study the genetic diversity of flue-cured tobacco. Tobacco is an allotetraploid that contains 24 pairs of chromosomes [35]. To guarantee an equal number of primers for each chromosome, 48 pairs of SSR markers were selected. In other words, each chromosome contained two pairs of SSR markers. We then analyzed the potential correlations between the intervarietal genetic relationships revealed by the 48 SSR marker pairs in addition to those that were revealed by the original 91 SSR marker pairs. The genetic relationships revealed by the two SSR marker sets were consistent with each other, which further justified the use of only 48 pairs of SSR markers. This is close to the minimum number of SSR markers for rice, which varies from 50 to 70 [33]. Rice and wild rice in particular present significantly higher genetic diversity than tobacco, further indicating that 48 pairs of SSR markers are sufficient to study the genetic diversity of tobacco varieties.

In this study, the genetic fingerprint of standard flue-cured tobacco varieties was constructed by using 48 pairs of SSR markers. As such, the 48 SSRs are core markers that can be applied to molecular-based DUS testing of flue-cured tobacco varieties. From YC/T 369–2010 [5], the 33 varieties evaluated in this study were distinct and presented a minimum difference of 4 SSR markers. Therefore, when using the aforementioned 48 SSR markers to evaluate the distinctness of candidate varieties, the number of distinct markers among the candidate and control varieties must be either 4 or more; otherwise, the candidate and control varieties are similar and field phenotypic identification should be performed according to YC /T 369–2010 [5] or TG/195/1 [4]. Thus, field experiments are only needed for similar varieties, which will greatly improve the efficiency of DUS testing.

Currently, single nucleotide polymorphism (SNP) markers have become an attractive alternative to SSR markers given the progress in genomic research and high-throughput sequencing [36, 37]. Although the diversity level of single locus is lower than that of SSR marker, and more loci are required to equal SSR detection effect, as dimorphic markers, SNPs can provide objective and readily distinguishable results that are well suited for DUS testing. Research on crop variety identification using SNPs has already been conducted [38–42]. Next, we intend to resequence the 33 varieties used in this study to find stable and reliable SNP loci and to explore SNP-based tobacco DUS testing.

## Conclusion

We used 48 SSR markers to generate the genetic fingerprints of standard flue-cured tobacco varieties commonly used in DUS testing. The 48 SSRs were considered to be core SSR markers that can be used for future flue-cured tobacco DUS testing. Molecular-based SSR DUS testing will improve the detection efficiency of traditional DUS testing methods while reducing costs. This method is also crucial for guaranteeing objectivity, fairness, and accuracy with regard to the verification of new varieties.

## Methods

### Plant materials

The 33 standard flue-cured tobacco varieties (Table 2) commonly used in DUS testing were provided by the National Crop Germplasm Resources Infrastructure (NCGRI; Tobacco, Qingdao).

**Table 2:** The 33 studied varieties and their typical characteristics

Code	Variety	Type	Typical characteristics
SV01	NC82	I	elliptical leaf shape
SV02	XHJ 1025	L	fewer leaves, susceptible to tobacco black shank disease
SV03	G28	I	low ratio of leaf length-to-width, susceptible to CMV
SV04	Zhongyan 90	B	wrinkled leaf surface, buckling leaf margins
SV05	Zhongyan 15	B	concentrated inflorescence
SV06	Coker 176	I	larger auricles, moderate tips of corolla
SV07	NC89	I	flat foliage, green leaf color
SV08	K326	I	fewer axillary buds, wavy leaf margin, light red flower color,
SV09	Zhongyan 100	B	wavy leaf margins, short flowers
SV10	HHDJY	B	flat foliage, little corolla, red flowers
SV11	Ge 3	B	resistance to tobacco black shank disease, moderate resistance to TMV
SV12	JYH	B	resistance to tobacco brown spot disease
SV13	Zhongyan 103	B	buckling leaf margins
SV14	G140	I	susceptible to tobacco brown spot disease and TMV
SV15	T.I.245	I	resistance to CMV
SV16	Coker139	I	fewer axillary buds, turbinate inflorescences
SV17	K149	I	narrow leaf width, light green leaf color
SV18	JX 6007	B	moderate resistance to tobacco black shank disease
SV19	CBH	L	longer leaves, large ratio of leaf length-to-width, obtuse leaf tips, susceptible to tobacco bacterial wilt
SV20	Ge 5	B	very tall plants with many leaves
SV21	NC-22-NF	I	very tall plants with many leaves, short leaf length, late flowering.
SV22	Wanye	I	petiolate, wide ovoid leaf shape
SV23	DB 101	I	resistance to tobacco bacterial wilt
SV24	NC-agz	I	dwarf plants
SV25	NC27NF	I	tall plants with many leaves, late flowering
SV26	Coker 254	I	light green main stem color
SV27	NC86	I	dark green main stem and leaf color
SV28	MN373	I	small auricles, early flowering.
SV29	B. L. Orinoco	I	long ovoid leaf shape
SV30	XHJ 5209	L	ovoid leaf shape
SV31	Coker371Gold	I	concentrated inflorescences, wavy leaf margins, wrinkled leaf surface
SV32	TGBHKY	I	white flower color
SV33	Guiyan 11	B	spherical inflorescences

^a^I, B, L indicate introduced, domestic, and local varieties, respectively

### SSR markers

A total of 270 polymorphic SSR markers were selected from a previous study [23, 43].

### DNA extraction

DNA extraction of 33 varieties was carried out with the following steps. Firstly, one hundred milligrams of the fresh leaves were ground in liquid nitrogen, and placed in a 2-mL EP tube. Secondly, 800 μL of SLS extracting solution (0.1 mol/L Tris-HCl, 0.2 mol/L EDTA, 0.1 mol/L NaCl, 10 g/L Sodium Lauroyl Sareosine, pH 8.0) was added, and the tube was shaken for 5 min. Thirdly, 800 μL of an isometric phenol: chloroform: isoamyl alcohol (25: 24: 1) mixture was added, followed by shaking for 5 min, and centrifugation at 12000 rpm for 10 min. Fourthly, 600 μL of the supernatant was transferred to a new 1.5-ml centrifuge tube and isometric precooled isopropyl alcohol (− 20 °C) was added for DNA precipitation. Next, the sample was centrifuged at 12000 rpm for 10 min, and the supernatant was removed, followed by a wash with 75% ethyl alcohol and a rinse with pure alcohol. Lastly, the sample was dried on a sterile bench for 30 to 60 min until no alcohol residue remained, and the sample was suspended in 100–200 μL of ddH_2_O.

### Polymerase chain reaction (PCR) amplification and electrophoresis

PCR amplification and polyacrylamide gel electrophoresis were conducted following the methods reported in previous studies [23, 43]. NaOH silver staining [44] was used for dyeing and developing the polyacrylamide gels.

### Data analysis

The amplified SSR band patterns were recorded in Excel 2013 (Microsoft Corp., Redmond, USA) using a binary (0–1) data format. The data were then converted by DataFormater [45] into input files for PowerMarker v. 3.25 [46], NtSys v. 2.10e [47], and Popgene v. 1.32 [48]. The average PIC was calculated using PowerMarker v. 3.25. Both H and I were calculated using PopGene v. 1.32. NtSys v. 2.10e was used to calculate genetic distances and to draw the UPGMA clustering tree. The software SPSS v. 22 [49] was used to generate boxplots and scatter plots and to perform correlation analysis. The random sampling of 1–90 markers was repeated 50 times for each marker number and the average PIC values were calculated. A Python (2.7) script was used for the random sampling experiment and for the statistical analysis of PIC values variation between samples. Other data analyses and the illustration of genetic fingerprints were carried out in Excel 2013.

## Supplementary information

**Additional file 1: Table S1**. The details of the 91 SSR markers studied in this research. The information of linkage group, genetic position, and sequence of primers came from reference [50].

**Additional file 2: Figure S1**. The electrophoretic image of SSR marker PT50136 (on the right side of the photo).

## Data Availability

The datasets used and/or analyzed during the current study are available from the corresponding author upon reasonable request (Min Ren, renmin@caas.cn).

## Abbreviations

CV: Coefficient of variation
DUS: Distinctness, uniformity, and stability
H: Nei index
I: Shannon information index
NCGRI: National Crop Germplasm Resources Infrastructure
PCR: Polymerase chain reaction
PIC: Polymorphic information content
SLS: Sodium lauroyl sarcosinate
SNP: Single nucleotide polymorphism
SSR: Simple sequence repeats
UPGMA: Unweighted pair group method with arithmetic mean
UPOV: International Union for the Protection of New Varieties of Plants
