# Supplementary material for: Genetic diversity and fingerprinting of 33 standard flue-cured tobacco varieties for use in distinctness, uniformity, and stability testing
Source: BMC Plant Biol. 2020 Aug 17;20:378. doi: 10.1186/s12870-020-02596-w (PMC7433079; doi:10.1186/s12870-020-02596-w)
Supplement: Supplementary file 1 — Additional file 1: Table S1. The details of the 91 SSR markers studied in this research. The information of linkage group, genetic position, and sequence of primers came from reference [50]. [file 12870_2020_2596_MOESM1_ESM.docx]

**Supplementary information**

**Additional file 1**

**Table S1** The details of the 91 SSR markers studied in this research. The information of linkage group, genetic position, and sequence of primers came from reference [50].

| Marker | Group | Position (cM) | Forward primer (5'→3') | Reverse primer (5'→3') | Number of alleles | PIC |
| --- | --- | --- | --- | --- | --- | --- |
| PT54339^a^ | 1 | 6.654 | CCCGTGTGATAAACATTGATTG | CCCGCCAGAAACTATTTACA | 3 | 0.2944 |
| PT1199 | 1 | 29.185 | TCGGGTCGTTACAACTGATG | CCACGTCATTCGGAGTTGTT | 3 | 0.2509 |
| PT60177 | 1 | 68.842 | TCACTCCCATCATCTCACCA | GGGACAGAGAGGTTTGGGA | 4 | 0.5641 |
| PT54593 | 1 | 88.84 | GCCGTTAAGGCTTTGTCAAT | TTCGCACGTCATTGTAGTCA | 4 | 0.2257 |
| PT30087 | 1 | 91.019 | CTTCTTCCTAAGCCGAGGGT | TTGATGATAGAACGCAACTCG | 3 | 0.4083 |
| PT51015 | 1 | 91.567 | TGTTGCGTGTTTACTTTGGG | AAGCCTCGAATAGGTGAGGA | 2 | 0.2241 |
| PT50063 | 1 | 91.567 | AAACTTAGGCCAAACAGGCTC | CACTTGCCTTTGTCTCGTCA | 4 | 0.2599 |
| PT54759 | 1 | 93.789 | CGTGCTGTTGAATATCCCATT | ATTTGGACAGCACAAGCTCC | 2 | 0.2241 |
| PT54587 | 1 | 94.058 | AGAACACCCTTCTCCAGCAA | TTCCTAAAGCTTTGACAGTTAAATAAA | 4 | 0.3819 |
| PT50862^a^ | 1 | 98.775 | ATGCGCTATGTTGGCTTCTT | TCAAAGTGCAATGTGTTCGC | 3 | 0.5107 |
| PT60826 | 1 | 98.775 | CAGTGGCCAATTCAAATTCA | CCAGGTTCATCTGACTCGGT | 2 | 0.2392 |
| PT51438 | 1 | 107.015 | TTGCTCTTGTCTCCTCCGTT | TCTTGACAATTACCCACGTACAA | 4 | 0.3933 |
| PT53216^a^ | 2 | 0 | TCCAAACTCTTATCCCTGATCC | CACCTACCCTGTCGCTGTTT | 3 | 0.1614 |
| PT52432^a^ | 2 | 53.864 | CAAAGTCCATACGCGTCCTT | GGCGGTACAGAAGATGAGGA | 3 | 0.2809 |
| PT53362^a^ | 3 | 45.272 | TTGAACTCGTTAAGTTTAGATAGCG | GCTGATTTGCAACGCAGTAA | 3 | 0.3921 |
| PT61116 | 3 | 85.215 | GGACCAATTTCTTCCATTGC | GGAAGCAAGAAGGAGACAGC | 5 | 0.3483 |
| PT52706 | 3 | 133.148 | AGACAGCGAAGAAGCAGAGG | GTCAGCCATCCTAGCGACTC | 2 | 0.3260 |
| PT53808 | 3 | 152.31 | CATGAGGTTTGAAGGCAACA | TGGAGCTTTGAGTGCGAAAT | 3 | 0.4212 |
| PT60080^a^ | 3 | 179.15 | CTACGCAGAATCCAATTCCA | CATTAGGCCATAGCCATCCA | 4 | 0.3400 |
| PT53970^a^ | 4 | 54.25 | TCATTGGTTCCTCGTCCTTT | AAGACAACTCCTTCACCATTCA | 2 | 0.2241 |
| PT51682^a^ | 4 | 76.882 | GGTTCAACTTCCAACTATGACATC | TGGTGATAAGTAGGACTGCTTGG | 4 | 0.6243 |
| PT60181 | 5 | 1.399 | TCACAATGTGGTGGACTTCTG | AGAGCAGATCTGATGCGAGT | 2 | 0.1516 |
| PT51072^a^ | 5 | 67.324 | CGATCCCGATGACACAATTT | CTTCCATTGTTCAAACTCGC | 3 | 0.3481 |
| PT61414^a^ | 5 | 79.527 | AAAGAAAGGAGGCATGCAAA | CAATGACTAATAGAATCGGTTACAGG | 3 | 0.4086 |
| PT54627 | 5 | 100.225 | GGCAATCGCGTGTTAGAAGT | TGCTCTTCGTAGTGATCTGCC | 4 | 0.2137 |
| PT60038^a^ | 6 | 11.242 | CATCACCTTTCTCATTTCCACA | GCTGACAAGTGATGATTCTACCA | 3 | 0.4442 |
| PT50434^a^ | 6 | 96.873 | GCCGTCAGCAATAAGACTCC | GTTCGGATCCGTGACCTAAA | 4 | 0.5906 |
| PT50001 | 7 | 22.855 | TGAGCAGAACTGACACGGAG | ATCGGAGGCATCCTCAGTTA | 3 | 0.3194 |
| PT50599^a^ | 7 | 37.82 | GCGAACCTTTGAACCAGTCT | AAACGCCTAGGCAGAACTCA | 5 | 0.3126 |
| PT52835 | 7 | 55.73 | AATGCAATACCAGTAGGCCG | ATTCCTCGCCCGTGTAAATA | 3 | 0.2639 |
| PT60435^a^ | 7 | 115.365 | AAACTCGGTGGGAGAGTGAG | CCCTTCTTGTTCGGTAAAGC | 4 | 0.4375 |
| PT52940 | 7 | 134.635 | TCTTAGCCGTCCATCTCTGC | CACAGGACAATTTGTTGCAGTT | 3 | 0.3275 |
| PT50668^a^ | 8 | 1.099 | TCCAATTACATTTGGAGCTTCTC | TCCTGGATTTGTCACTGCAC | 2 | 0.1516 |
| PT61279^a^ | 8 | 120.597 | CTCACACCCATTTGTTACCG | GGCGACAATGTTGTTGTGTC | 3 | 0.2747 |
| PT50280^a^ | 9 | 4.97 | CACAATATTCATGCCAAGGG | ATCGAAGAGGACAGGGACAA | 4 | 0.4161 |
| PT60917^a^ | 9 | 38.417 | CAGGAGACTCCATAAATTCACTCTT | AGCATGGTTGCTATTGGCTG | 2 | 0.1903 |
| PT51144^a^ | 10 | 24.533 | ACCGACAACACACGATTGTA | GCTTGTCGCTTAGCTTAACCAT | 4 | 0.3933 |
| PT54061^a^ | 10 | 46.603 | CAACGTGTGCCGAAAGACTA | TTGGGAACGTGAGGGATATG | 2 | 0.1516 |
| PT51398^a^ | 11 | 2.208 | TCCAAAGAAATTCAATCCGC | AGCAAATTCCTCACCTTTGC | 4 | 0.2841 |
| PT54027^a^ | 11 | 50.95 | GGATCTGCTCATTTCCTATGG | CAAGGCAGCTAAGCCTAACAA | 2 | 0.2583 |
| PT60934^a^ | 12 | 55.632 | CACACATCAGCTGCACATTG | CCTCACGAAGCATTCCATAAA | 5 | 0.6024 |
| PT51896^a^ | 12 | 130.061 | CTTCCCTCTGTAACAACGCC | AACAGTTTCAATTATAACACTGCCA | 4 | 0.3359 |
| PT53568^a^ | 13 | 38.293 | CGTTTCTCTTCCAATTAACAGC | ACGTCATCAATGGCATCAAA | 4 | 0.6499 |
| PT60844^a^ | 13 | 75.285 | ATTCTTCAGGCAAACGATGG | TTGCAAGATGAGAAGAGGGAA | 4 | 0.2554 |
| PT61499^a^ | 14 | 0 | ATCGACCGGGCGAAATTA | GCCCAAGATTGTACTCTCCTCA | 4 | 0.4577 |
| PT54448^a^ | 14 | 42.076 | CTGCTCTTGAGAATGTGCCA | TTTACTCCAAGAGCAACTTTGAA | 2 | 0.3180 |
| PT52919 | 15 | 54.437 | GCCTCCATCAAAGGTCACAT | GAGTCGGGCAATAGAGACGA | 2 | 0.2743 |
| PT30201^a^ | 15 | 64.96 | GGGTTGGCCAATATGTGTACTT | GGCTTCTCCAACATCATCAAA | 5 | 0.3483 |
| PT54772^a^ | 15 | 108.802 | TCAGCTCCTCCTTTCTCCTG | CTTACAAATTTCGCCTCGGT | 3 | 0.3030 |
| PT20275^a^ | 16 | 23.212 | GTTCTATTTGATCGCCCC | AACAGCACCAACAGCATT | 3 | 0.4683 |
| PT50624 | 16 | 47.76 | ATAGGGCCTAAGTGCTGGCT | CTGAAGGCTTGTTTCGGTGT | 2 | 0.2241 |
| PT30302 | 16 | 69.375 | CCTTCCTAACCTCAGCTGGAA | TATGCCAATGCTTCTTGTGG | 4 | 0.5690 |
| PT55150^a^ | 16 | 76.1 | TAACAGATAATCTACAACGCTATGTTT | TGGGATGTAACGAAAGGAGA | 4 | 0.3756 |
| PT50748^a^ | 17 | 12.738 | CAGATATTAAACCAGCCTATGCAA | AGTCACAACGCAGCTAGCAA | 4 | 0.6191 |
| PT50693^a^ | 17 | 20.14 | TCATGAGAGGCAGACAGTGTT | TTGTGATGTTGTAATCCTGTTGG | 4 | 0.6557 |
| PT51333 | 17 | 21.227 | GCACCTTTGGTTATCCGACA | TGCTTTAAGTCATGTACCAAATTGA | 5 | 0.6211 |
| PT51562 | 17 | 46.431 | CATTCTTGGCAGGCATTGTT | TTTCGAGTTTGAGAAACTGTCA | 3 | 0.3220 |
| PT61564 | 17 | 102.013 | GCCAACATGACTCTGACGAA | TCTGTGTGTGCGGATTGATT | 3 | 0.3855 |
| PT53732 | 17 | 109.486 | TCCACAATCTTCAACTGCAAA | TTTCATGGGAGAGAAGGTGC | 2 | 0.2999 |
| PT51378 | 17 | 111.136 | ATGTCGGGAAGATTCCACAG | ACAACCAGCTAAATGTGCCC | 6 | 0.4632 |
| PT51059^a^ | 18 | 15.309 | CAAACCAGCGCATCTGTCTA | CACCAGAACCCGCTAGTCTC | 3 | 0.1125 |
| PT60742^a^ | 18 | 40.185 | TGAGTTCCATTCTAGGGCTGA | GCATTTGCAACAGAAGCGAT | 4 | 0.2137 |
| PT50500^a^ | 19 | 96.118 | ATCGGCTTCATAAGGCAAGA | ACCTTTGCAAGGAGGTTTCA | 3 | 0.3275 |
| PT54889^a^ | 19 | 106.147 | GGGTCTAAATAATCGCAATTGAA | CGCAGGTGGACTTAAAGAATC | 4 | 0.4707 |
| PT50700 | 19 | 107.793 | CGTCTGTCTCTTTCTGTAATTCCC | GAGGAAGGGACAAGGAAAGG | 4 | 0.1883 |
| PT30180 | 19 | 117.284 | CAGCTCGAACATGGAGAATC | GATGATTCAAGTCTCACTCACCA | 2 | 0.1074 |
| PT50298^a^ | 20 | 78.404 | CCACAGCATGTAGACAACGG | AGTCGAAGTTCAAACAGTTGGT | 3 | 0.4543 |
| PT30421^a^ | 20 | 93.545 | CGTACCCTGAATGCCATCTT | ATGCAGCGTTTCAGGAATTT | 2 | 0.2634 |
| PT50245 | 21 | 29.291 | TCTAATTCAACTTTGGATCCATTT | CTTCTGCAGTTAGCCACCCT | 4 | 0.5098 |
| PT51951^a^ | 21 | 41.966 | GCCCGAACAAATGTGTATAGAA | TGCTGACTTTCATTCCTCGTT | 5 | 0.4390 |
| PT51289^a^ | 21 | 49.7 | GAGTTGTGGCCAAGTAGCCT | GGCTTTAGCCAAACGCTCTA | 3 | 0.4482 |
| PT51152^a^ | 22 | 96.25 | GCGCAGTATTTAGCCCAACT | CATTCCATCACAAGCTTCCA | 5 | 0.4688 |
| PT52041^a^ | 22 | 142.487 | CGTGACGAAACATTGTTGGA | TTTGACGGGTATTTGAGTCTTT | 3 | 0.4415 |
| PT55187 | 23 | 2.491 | AATCAACGCGTCTTCTATTTATTATT | TCGTAGATCAAATGGATACGTAAA | 3 | 0.3714 |
| PT60990 | 23 | 4.978 | TCAACCCATAAAGCTGCTCC | AAAGAGACAAAGCAGGCACAA | 5 | 0.3090 |
| PT50062 | 23 | 16.858 | AGGATCGAAATGAGATGAGAAG | GCAATTTGCTCTCTCCAGTTG | 5 | 0.5617 |
| PT50336^a^ | 23 | 24.858 | AGTGACCATTAAGTATATGTCTAAGCC | GCGACCCGATTAGCTTCTAT | 2 | 0.2999 |
| PT52509 | 23 | 49.555 | TTGCCATTCCTTTATTTCCG | AAATAACAGAATCTCTGGCGAA | 3 | 0.4604 |
| PT20445 | 23 | 55.675 | CAAGAACAATGTGTCAACTGTGAA | ATGGCAAAATGTTGCATCTC | 4 | 0.4507 |
| PT50425 | 23 | 55.675 | CGGTGAGAATTAAATACCGTGG | TTGGTGTATTTCGTGCAAGC | 3 | 0.2331 |
| PT20192 | 23 | 59.797 | ATATTTGCTTCTGGGGCT | AAACCACGCGTACCACTA | 5 | 0.5907 |
| PT52156 | 23 | 60.339 | TGAGAATTCATATAACCGACCCA | TGCCTTCTTTCATACTGCCA | 2 | 0.3318 |
| PT54748 | 23 | 66.994 | TGCAACTACAAATTATCAAGAAGGA | CGGTCGGATCGTTACAGAAA | 2 | 0.3692 |
| PT53595 | 23 | 67.542 | CTTAGGACCCAAGAACCCAA | TCCATTCTTGTCCAAGGAGC | 3 | 0.3656 |
| PT50136^a^ | 23 | 69.205 | TTTGGGATGGTTCTGCAAAT | GAAACTTTCCTAACCGCGAA | 2 | 0.3692 |
| PT53796 | 23 | 81.966 | AAGGGCAGATACGACCATTT | CACAAATTGATCACCCTCCC | 2 | 0.3494 |
| PT30380 | 23 | 98.311 | AACGAGTGTAAACATGCTCCC | CCACCAGCACAATAATGAACA | 5 | 0.4869 |
| PT53915 | 23 | 123.065 | GCCACGTGTTAAGACCGAAT | AGCATCGAGTGTATCATCAAACTA | 3 | 0.1614 |
| PT52080 | 24 | 14.543 | TCTGTTGCTATTTATTGCTTTGTCA | AACATGTTGTGGAGTGCAGC | 3 | 0.3030 |
| PT50541^a^ | 24 | 40.707 | GGCTTGACGCTGTATCAGG | CATGACTCACACTTCGCCAC | 5 | 0.3825 |
| PT52828^a^ | 24 | 69.018 | TGAATTATGCGGTGCACATT | ATATTCCAACGTGGGCTGAA | 2 | 0.3635 |

^a^  Indicated that this marker was one of the 48 SSR markers which were used to construct genetic fingerprint in this study.
