# Supplementary figures and images for: Genetic diversity and fingerprinting of 33 standard flue-cured tobacco varieties for use in distinctness, uniformity, and stability testing
Source: BMC Plant Biol. 2020 Aug 17;20:378. doi: 10.1186/s12870-020-02596-w (PMC7433079; doi:10.1186/s12870-020-02596-w)

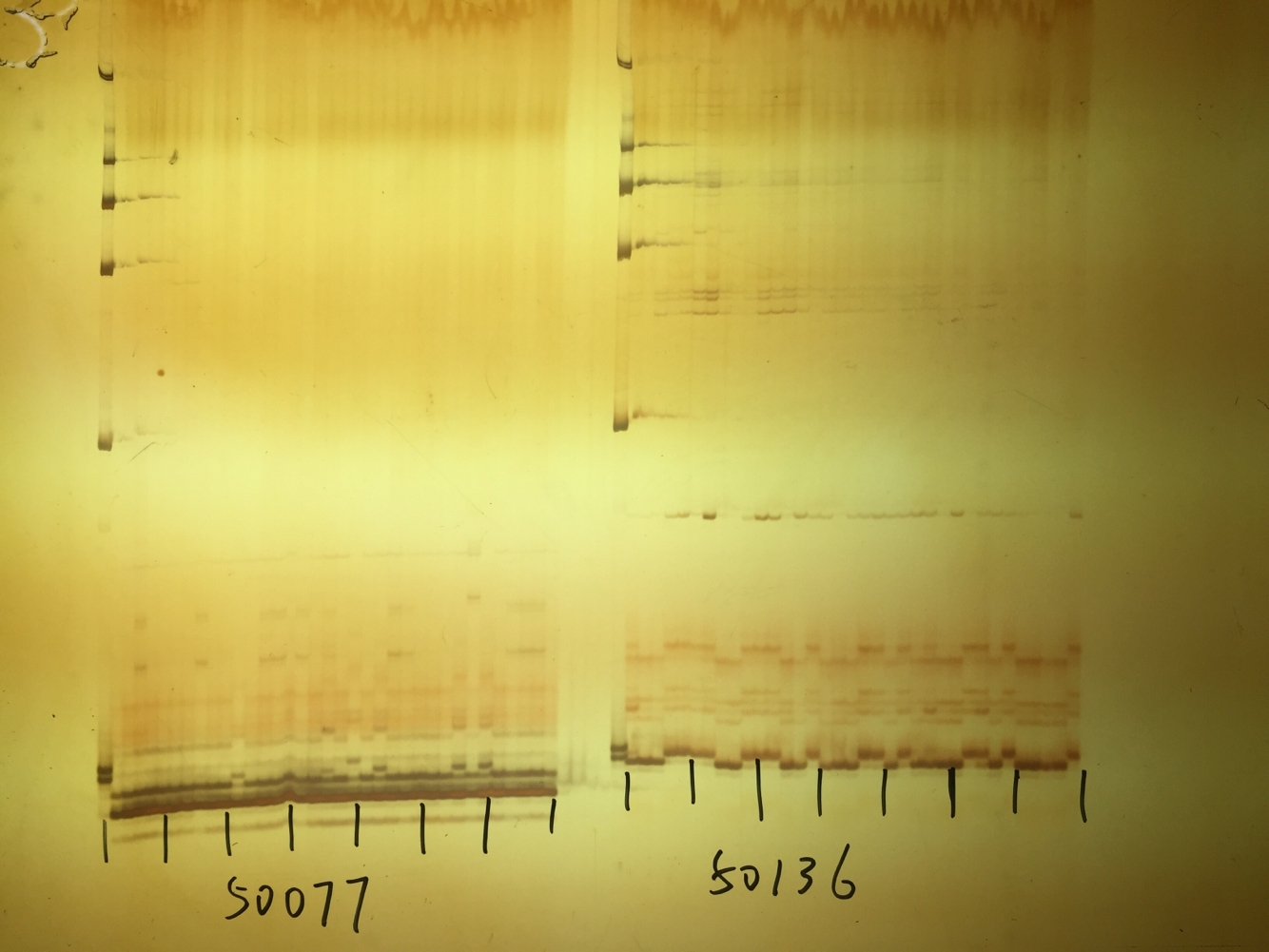

Supplement: Supplementary file 2 — Additional file 2: Figure S1. The electrophoretic image of SSR marker PT50136 (on the right side of the photo). [file 12870_2020_2596_MOESM2_ESM.png]
